# Musculo-skeletal pain among 40- and 45-year olds in Oslo: differences between two socioeconomically contrasting areas, and their possible explanations

**DOI:** 10.1186/1475-9276-3-10

**Published:** 2004-10-19

**Authors:** Mette Brekke, Per Hjortdahl

**Affiliations:** 1 Department of General Practice and Community Medicine, University of Oslo, Box 1130 Blindern, N-0317 Oslo, Norway; 2 Per Hjortdahl, Department of General Practice and Community Medicine, University of Oslo, Box 1130 Blindern, N-0317 Oslo, Norway

**Keywords:** population survey, social inequalities in health, muskulo-skeletal disorders, mental health, immigrants

## Abstract

**Background:**

The objective of the study was to compare the prevalence and severity of musculo-skeletal pain between two socioeconomically contrasting areas in Oslo, Norway, and to explore possible explanatory factors.

**Methods:**

Questionnaire survey, carried out as part of The Oslo Health Study in 2000–2001. Data from 821 persons (40 and 45 year old) living in a less affluent inner city area (called east) were compared with 854 persons living in an affluent area of the city (called west). Bivariate comparisons (chi square test) and multiple regression analyses were performed to investigate differences between the samples.

**Results:**

61 % in east and 56 % in west (p < 0.05) reported pain/stiffness in muscles/joints during the last four weeks. 30 % in east versus 19 % in west (p < 0.001) reported extensive pain. The between area difference in extensive pain was partially explained by physical inactivity, mental health problems and being of non-Western origin.

**Conclusion:**

Musculo-skeletal pain is reported by 55–60 % of middle aged persons in Oslo during a four week period, and must be considered a normal phenomenon. Poor social conditions, inactivity, mental health problems and being an immigrant imply increased risk of more severe symptoms with a concomitant demand of health care.

## Background

In affluent societies like Norway, living conditions as well as general health status have improved during the last decades. In spite of this, social health inequities still exist, and recent analyses from Oslo even indicate an *increase *during the last 30 years [1]. Life expectancy for men living in the least affluent city area is 69 years, compared to 76 for men in the most affluent area [2]. Well-known risk factors like smoking, physical inactivity and overweight, as well as the incidence of atherosclerotic disease and several forms of cancer show similar correlation [3].

Some claim that future research on this topic should concentrate exclusively on interventions [4]. In Great Britain as well as Holland this has been highlighted for some time [5], and in Holland a national strategy for tackling health inequities has been developed [6]. Nevertheless, we need to continuously keep an eye on trends, as well as on causal and maintaining factors. And even if we have ample data on socioeconomic inequity regarding mortality and morbidity, we know far less about the dimensions of disease severity and patients' coping ability, in Oslo as elsewhere [7]. The aim of the present study was to investigate differences in prevalence and severity of musculo-skeletal pain between middle aged inhabitants of two socioeconomically contrasting areas in Oslo, based on a recent and comprehensive data collection, and to examine some possible explanatory factors. We chose to study musculoskeletal pain, as this is a major cause of disability in the industrialised world [8]. In Norway, musculoskeletal pain generates 15–20 % of consultations in primary care, and is one of the main reasons for sick leave and social security [9].

## Methods

The data collection was part of the Oslo Health Study, a joint collaboration between the Oslo City Council, the University of Oslo and the Norwegian Institute of Public Health, which was conducted from May 2000 to September 2001. All residents born in 1924/25, 1940/41, 1955, 1960 and 1970 (n = 41353) received the three-page main questionnaire by mail, as an invitation to participate in a health screening. At the screening station a simple clinical examination and a blood test were performed, and the questionnaire was handed in. Two supplementary questionnaires were given out: one identical for all age groups, and one in four different versions. Participants were asked to fill in the supplementary questionnaires at home and return them by mail. Two reminders were sent to non-respondents. An overview of all topics covered in the questionnaires (in English) can be obtained from .

In the present study we analysed data from persons born in 1955 and 1960, who lived either in the inner eastern part of Oslo or in the outer western part (see below). We used data from the main questionnaire as well as from the age specific supplementary questionnaire.

The variables included from the main questionnaire were: marital status, educational level, employment status, disability pension, social assistance, country of origin, physical exercise, alcohol intake, smoking habits, general health status, mental health problems, and musculo-skeletal disorders. Country of origin was recoded as Western (Western Europe, North America, Australia) or non-Western (Eastern Europe, North Africa, Sub-Saharan Africa, Middle East, Indian subcontinent, Eastern Asia, Pacific, Middle America, South America) [10].

Mental health problems were assessed by the following question: *Below is a list of various problems: Have you suffered from any of the following during the last week, including today? Put a cross for every problem*. Choices: Not troubled, slightly troubled, quite a lot troubled, much troubled (values 1–4). The values were summarised and divided by the number of answers, and a mean value of 1,85 or more was used as a marker of mental health problems [10].

Musculo-skeletal pain was explored by the following question: *Have you suffered from pain and/or stiffness in muscles and joints in the course of the last four weeks? *Choices: Not troubled, somewhat troubled, very troubled (values 1–3) for the alternatives neck/shoulders, arms/hands, upper back, lower back, hips/legs/feet and elsewhere. The values were summarised and divided by the number of answers. A mean value of 2 or more was used as an indicator of extensive pain/stiffness in muscles or joints [10].

The variables from the age specific supplementary questionnaire included were: own income, household income, muscular pain/stiffness last 4 weeks, duration of muscular pain/stiffness, satisfaction with health care, and belief in own coping ability.

### The east and west areas

Oslo's local authority districts can be ranked according to: level of income, education, employment, disability pension, housing standard, number of non-western immigrants, and mortality [11,12]. According to this ranking, three districts in the western part of the city are on top, indicating the best socioeconomic conditions. These are the districts Vindern, Røa and Ullern, here called west. Three districts in the inner eastern part take on the least favourable positions: Sagene-Torshov, Grünerløkka-Sofienberg and Gamle Oslo, here called east. Per January 1^st ^2000, west had 67296 inhabitants and east 80 668. (Since the study was done, the city of Oslo has reorganized the local authority districts. Vindern and Røa are joined under the name Vestre Aker, and the names of two others are changed to Sagene and Grünerløkka). We have chosen to compare these two areas, because they are strongly contrasted regarding living conditions.

### Statistical analyses

Statistical analyses were performed using SPSS version 11.0. Bivariate comparisons of categorical variables were examined by the chi square test. Multiple regression analyses (stepwise) were performed to estimate the explanatory power of independent variables. A 5 % level of significance was chosen.

## Results

The main questionnaire was completed by 821 forty- and 45 year olds living in east (50.7 % women) and 854 living in west (62.9% women), corresponding to a response rate of 39.0 % in east and 43.9 % in west. Some returned the questionnaire without attending the health screening, meaning that 1348 persons completed the supplementary questionnaires.

There was no significant difference regarding full time employment, and frequent use of alcohol was more common in west. For all other socioeconomic and lifestyle variables, as well as general and mental health, east came out poorer (Table 1).

**Table 1:** Oslo Health Study 2000–2001, 40- and 45- year olds Demographic variables, lifestyle, and self-reported health in east, west, and the whole city (percent).

	East	West	City
Education =< 9 years	14.0	2.0	8.3
Education > 12 years	57.3	87.8	65.8
Single status	15.4	8.0	9.5
Employment full time	67.4	71.2^1^	72.8
Disability pension	10.5	2.7	5.6
Social benefit	6.9	0.5	2.6
Own income < 200 000	30.2	19.6	24.6
Own income > 400 000	8.5	30.0	20.0
Household income < 200000	21.0	4.0	12.0
Household income > 500000	22.7	66.9	46.2
**Non-western country of origin**	24.5	5.4	16.8
No hard exercise	35.1	19.5	28.7
Alcohol at least once a week	49.4	66.9	52.9
Daily smoking	41.4	22.4	32.5
Less than good health	29.3	11.7	21.2
Mental health problems	23.1	9.4	12

**^1^Not significant difference east versus west **All other variables: significant difference, p < 0.001 (chi square test)

The proportion having experienced muscular pain/stiffness during the last four weeks, being very troubled by muscular pain/stiffness in various body parts, or reporting extensive pain/stiffness was higher in east. No difference was found regarding pain duration. Participants in west were more satisfied with health care and more confident in own coping ability (Table 2).

**Table 2:** Oslo Health Study 2000–2001, 40- and 45- year olds Musculo-skeletal disorders in east, west, and the whole city (percent).

	East	West	**City**
Pain/stiffness in muscles/joints last four weeks	61.4	55.9*	58.4
Very troubled by pain/stiffness in arms/hands	6.7	4.0*	5.3
Very troubled by pain/stiffness in neck/shoulders	13.6	7.5^++^	10.5
Very troubled by pain/stiffness in upper back	7.6	**3.1**^++^	5.3
Very troubled by pain/stiffness in lower back	11.4	4.8^++^	8.0
Very troubled by pain/stiffness in hips/legs/feet	10.6	4.8^++^	7.6
Very troubled by pain/stiffness elsewhere	3.8	1.3^+^	2.4
Extensive pain/stiffness in muscles and/or joints	30.0	**18.9**^++^	-
Duration > 3 years	44.2	42.5	43.4
Satisfied with health care (quite satisfied/very satisfied)	28.5	38.9^+^	32.2
Confident in coping ability (quite sure/very sure)	74.9	86.2^++^	76.7

* significant difference east versus west (chi square test) p < 0.05

^+ ^p < 0.01

^++ ^p < 0.001

Female gender, living in east, low education, low own income, non-Western country of origin, no hard exercise and mental health problems were all correlated to extensive muscular pain/stiffness (Table 3, left column). Female gender, no exercise, non-Western origin and mental health problems still implied increased risk of extensive muscular pain/stiffness when the other variables were adjusted for. Low education and living in east no longer showed an independent correlation with extensive pain/stiffness after adjustment (Table 3, right column).

**Table 3:** Oslo Health Study 2000–2001, 40- and 45- year olds living in areas east and west Odds ratio for much pain/stiffness in muscles and/or joints, related to demographic variables, lifestyle and mental distress. Logistic regression analyses.

	Odds ratio (95 % CI), unadjusted	Odds ratio (95 % CI), adjusted^1^
Female sex	1.45 (1.15 – 1.83)	1.85 (1.40 – 2.45)
Single status	1.02 (0.82 – 1.29)	
Area east	1.88 (1.49 – 2.38)	1.22 (0.92 – 1.62)
Low education	2.11 (1.43 – 3.16)	1.09 (0.67 – 1.78)
Low income	1.34 (1.01 – 1.79)	
Low household income	1.1 (0.72 – 1.68)	
Non-western country of origin	4.41 (3.25 – 5.99)	3.17 (2.17 – 4.63)
Daily smoking	0.81 (0.64 – 1.04)	
No hard exercise	2.00 (1.55 – 2.59)	1.37 (1.01 – 1.85)
Mental health problems	3.87 (2.92 – 5.14)	2.70 (1.94 – 3.76)

^1^Variables included into analyses: sex, area, education, country of origin, exercise, mental health problems.

We performed the logistic regression analyses for men and women separately (data not shown). Non-western origin was the most important predictor of extensive pain/stiffness in men (OR 3.36, 1.93 – 5.83) and mental health problems in women (OR 3.04, 95 % CI 1.99 – 4.66). When we performed the analyses for respondents of Western and non-Western origin separately (data not shown), mental health problems were the most important independent predictor for extensive pain/stiffness for both groups (OR 2.87, 95 % CI 1.97 – 4.19 for Western, OR 2.2, 1.1 – 4.5 for non-Western).

## Discussion

In both areas around 60 % reported pain/stiffness in muscles/joints during the previous four weeks: 61.4 % in east and 55.9 % in west, a statistically significant difference of little clinical relevance. We do not know the prevalence among non-respondents, but as The Oslo Health Study implied a comprehensive data collection on many topics, it is unlikely that muscular problems in particular should influence response rate extensively. In a questionnaire survey we carried out in the same areas in 1994 (870 respondents in east, 892 in west, mean age around 40 years) approximately 55 % in both areas reported musculo-skeletal pain during the last four weeks [13]. In another Norwegian survey from 1991, only 15 % reported no muscular pain during the previous year, 58 % had experienced pain the last week, and 15 % reported pain every day during the previous year [9]. Periodic muscular pain or stiffness in one or more body regions should probably be considered a normal phenomenon among adults. Only when symptoms are strong, they imply disease and demand of health care [14].

It is thus important that the proportion reporting to be very troubled was significantly higher in east regarding all body regions. This might be due to a higher prevalence in east of specific musculo-skeletal diseases, like rheumatoid arthritis, fibromyalgia, etc. The Oslo Health Study asked about fibromyalgia and osteoporosis: In east 49 persons reported fibromyalgia and nine osteoporosis, compared to 19 and five in west. In previous studies we found no difference in prevalence of rheumatoid arthritis [15] or osteoarthrosis [13] between the two areas. The higher level of extensive pain in east corresponds, however, with our earlier results, as higher pain intensity, more widespread pain and higher disability scores were found among residents in east compared to west [13].

Blank and Diderichsen found social inequities in both frequency and intensity of a variety of common symptoms in a Swedish population [16]. Their results led to the hypothesis of "double suffering" also promoted by Eachus [7]: That lower classes both have more illnesses and experience these illnesses with greater intensity. Their lesser resources to cope with the consequences of disease also contribute to the suffering. Our present study lends support to this hypothesis: Physical and mental ill-health are more frequently reported in east, musculo-skeletal disorders are more common, the proportion reporting to be very troubled by pain is higher, and fewer respondents believe that they can continue their daily activities and fewer express satisfaction with health care.

The response rate in our material is low (39 % in east, 43.9 % in west). Total response rate in The Oslo Health Study was 46.5 % for 45- year olds, 43.7 % for 40- year olds and 46 % for all age groups. Non-attendance does not occur randomly. Analyses of the impact of self-selection on the Oslo Health Study have shown that the following sub-groups were under-represented among the attendees: unmarried or divorced, males, persons with low education, low income groups, receivers of disability benefit, inner city dwellers and those not born in Norway [17]. But when response rate is low, it also turns out that some healthy, highly educated and busy people have chosen not to participate [18]. We may suggest – but can not know for sure – that non-respondents in east belong mainly in the first group and in west mainly in the second. The implication would be that the differences observed between the areas would increase with increasing response rate.

We consider it a strength to use geographical area as a marker of socioeconomic position, and not for example individual education or income. Residential areas are distinct and easy to handle for authorities and politicians, and the majority of health care resources are allocated at area level. That inhabitants in affluent areas are healthier than in less attractive areas, is hardly a surprise, but which are the mechanisms behind the differences? There may be a certain amount of selection: The financial disadvantage of disabled people make it more likely that they live in poorer areas. In our material, far more people of non-western origin lived in east compared to west. As being of non-Western origin showed a strong independent correlation with severe muscular pain, this selection contributed significantly to the between area difference observed. A less healthy physical environment, less healthy lifestyle, and the psychological impact of being poorer than other people, are also possible explanatory factors [19]. Some authors have found that geographical variations in self-reported illness persist even after allowing for socio-structural individual characteristics [20,21]. This was not the case in our study, as area of living did not show any independent correlation with musculo-skeletal pain after adjustment for individual explanatory variables.

As our study is cross-sectional, causal interpretations cannot be made, we can only describe associations between socioeconomic measures and the health inequities observed. Several studies have shown that education and income can not explain the difference in self-reported health between socioeconomic contrasting areas [20-22]. Our results support this, and support the theory that psychological factors are important [23]. According to Wilkinson, socioeconomic inequality influences health through perception of place in the social hierarchy [24]. Such perceptions produce negative emotions like shame and distrust that are translated inside the body into poorer health via psycho-neuro-endocrine mechanisms [25].

## Conclusion

The present study shows that even in Norway today the perception and impact of a health problem (musculo-skeletal pain) is related to a person's socioeconomic situation. Self-reported health status is known to correlate with mortality, and it is a person's perceived health problems which influence the demand for health care. Significantly more persons living in a non-affluent area of Oslo reported extensive pain, compared to persons in an affluent area. Inactivity, poor mental health, and being a non-Western immigrant implied increase risk of severe symptoms.

## Competing interests

The authors declare that they have no competing interests.

## Authors' contributions

The two authors planned and carried out the data collection together. MB carried out the data analyses and drafted the manuscript. Boyh authors approved the final manuscript.
